# Potential natural immunization against atherosclerosis in hibernating bears

**DOI:** 10.1038/s41598-021-91679-1

**Published:** 2021-06-09

**Authors:** Shailesh Kumar Samal, Ole Fröbert, Jonas Kindberg, Peter Stenvinkel, Johan Frostegård

**Affiliations:** 1 Division of Immunology and Chronic Disease, Institute of Environmental Medicine, Karolinska Institutet, Stockholm, Sweden; 2 Department of Cardiology, Faculty of Health, Örebro University, Örebro, Sweden; 3 Norwegian Institute for Nature Research, 7485 Trondheim, Norway; 4 Department of Wildlife, Fish and Environmental Studies, Swedish University of Agricultural Sciences, 901 83 Umeå, Sweden; 5 Division of Renal Medicine, Department of Clinical Science, Intervention and Technology, Karolinska Institutet, Stockholm, Sweden

**Keywords:** Adaptive immunity, Antimicrobial responses, Innate immunity, Immunology, Cardiology, Cardiovascular biology

## Abstract

Brown bears (*Ursus arctos*) hibernate for 5–6 months during winter, but despite kidney insufficiency, dyslipidemia and inactivity they do not seem to develop atherosclerosis or cardiovascular disease (CVD). IgM antibodies against phosphorylcholine (anti-PC) and malondialdehyde (anti-MDA) are associated with less atherosclerosis, CVD and mortality in uremia in humans and have anti-inflammatory and other potentially protective properties. PC but not MDA is exposed on different types of microorganisms. We determine anti-PC and anti-MDA in brown bears in summer and winter. Paired serum samples from 12 free ranging Swedish brown bears were collected during hibernation in winter and during active state in summer and analyzed for IgM, IgG, IgG1/2 and IgA anti-PC and anti-MDA by enzyme linked immunosorbent assay (ELISA). When determined as arbitrary units (median set at 100 for summer samples), significantly raised levels were observed in winter for anti-PC subclasses and isotypes, and for IgA anti-PC the difference was striking; 100 IQR (85.9–107.9) vs 782.3, IQR (422.8–1586.0; p < 0.001). In contrast, subclasses and isotypes of anti-MDA were significantly lower in winter except IgA anti-MDA, which was not detectable. Anti-PCs are significantly raised during hibernation in brown bears; especially IgA anti-PC was strikingly high. In contrast, anti-MDA titers was decreased during hibernation. Our observation may represent natural immunization with microorganisms during a vulnerable period and could have therapeutic implications for prevention of atherosclerosis.

## Introduction

Free ranging brown bears (*Ursus arctos*) hibernate for 5–6 months during winter. Despite anuria and immobilization bears do not develop sarcopenia, cardiovascular disease (CVD) or osteoporosis^1^. Thus, bears can be seen as a translational model for sedentary life-style related diseases^2^. Although markedly elevated plasma lipids and obesity in fall are components of the hibernating bear phenotype their arteries show no signs of atherosclerosis, not even *in* early stages and they do not suffer from CVD^1,3–5^. This is in sharp contrast to the pro-atherogenic situation in humans with dyslipidemia, insulin resistance and chronic kidney disease (CKD)^6^. Thus, better understanding of protective mechanisms in hibernating bears may provide biomimetic information to identify novel treatment strategies for human life style diseases^7^. Atherosclerosis is an inflammatory condition, with dead cells, oxidized low density lipoprotein (OxLDL) and immune competent cells, producing mainly proinflammatory cytokines^8^.

PC and MDA are damage associated molecular patterns (DAMP), exposed on damaged and dead cells, and OxLDL^6,9^. *In addition,* phosphorylcholine (PC) is a pathogen associated molecular pattern (PAMP)^10^ exposed on bacteria like *S. pneumoniae* but also on nematodes, parasites and other microorganisms^6,8,11^. PC binds to proteins and carbohydrates in bacteria^6^ and may play a central role in OxLDL-induced immune activation in atherosclerosis^12^. Antibodies against PC (anti-PCs) of IgM isotype constitute about 5–10% of the circulating IgM pool of healthy adults and are relatively stable, though there may be a slight decrease with increasing age^6^. We previously reported that anti-PC but not anti-MDA is associated with protection in chronic lifestyle diseases associated with inflammageing^7^. IgM anti-PC is negatively associated with increased risk of stroke and myocardial infarction and also with atherosclerosis progress^6,13^. Animal experiments support a protective role of anti-PC in atherosclerosis^14^. Low IgM anti-PC is independent of classical risk factors for atherosclerosis and CVD with risk estimates comparable with smoking and hypertension^6^. These and similar findings have largely been confirmed and also extended to mortality in CKD and systemic rheumatic disease including SLE^6,13,15–25^. Also IgM anti-MDA is associated with protection in some conditions, such as SLE, CVD and uremia, though they have been less studied in humans than IgM anti-PC^26,27^. The role of IgG anti-MDA is less clear and since IgG2 anti-MDA is associated with increased mortality in uremia it may thus instead be negative^28^.

IgG1 and IgA anti-PC has similar properties as IgM anti-PC, associated with protection in atherosclerosis progress^29^. We have also reported that IgM and IgG1 anti-PC is associated with longevity in CKD^30^. Given the protection against arteriosclerosis in a dyslipidemic and uremic milieu we analyzed anti-PC *and anti-MDA* in paired summer (active state) and winter (hibernation) bear samples and report that anti-PC, especially IgA and IgG1 are strikingly high in hibernating bears *in contrast to anti-MDA*.

## Materials and methods

### Bears and collection of samples

Samples of blood were taken from 12 free-ranging sub-adult 2- to 3-year-old Eurasian brown bears, 9 females and 3 males equipped with a Global Positioning System (GPS) collar in Dalarna and Gävleborg Counties, Sweden, 2012–2014. Bears were captured during February–March and again during the summer active period (June). Details on sampling procedures have been presented elsewhere^4^. The field studies did not involve endangered or protected species. All animal handling and sampling was carried out under approval of the Swedish Ethical Committee on animal research (C212/9) and was in compliance with Swedish laws and regulations. The appropriate authority and ethical committee was “Djuretiska nämnden, Uppsala, Sweden”.

#### Antibody measurements

Bear antibody levels of IgM, IgG, IgG1, IgG2 and IgA anti-PC and anti MDA were determined by in-house ELISA as described previously^6,13,25,29,30^. The concentration of the antigen (used in each well was 10 μg/mL. Nunc Immuno microwell plates (Thermo Labsystems, Franklin Lakes, MA, USA) were coated with PC-Bovine Serum Albumin (PC-BSA) and MDA-Human serum albumin. Coated plates were incubated overnight at 4 °C. After four washings with wash buffer (1 × PBST), the plates were blocked with 2% BSA-Phosphate Buffered Saline for 1 h at room temperature. After similar washing steps serum samples were diluted for IgM, IgG, IgG1, IgG2 and IgA (1:100 for all) in 0.2% BSA-PBS and added at 100 μL/well. Plates were incubated at room temperature for 2 h and washed as described above. Biotin-conjugated goat anti-Human IgM, biotin-conjugated mouse anti-human IgG, biotin-conjugated mouse anti-human IgG1, biotin-conjugated mouse anti-human, IgG2, biotin-conjugated rabbit anti-human IgA (diluted 1:25,000, 1:80,000, 1:800, 1:15,000 and 1:15,000, respectively, in 1% BSA-PBS) was added at 100 μL/well and incubated at room temperature for 2 h. After four washings, the plates were incubated with horseradish peroxidase conjugated streptavidin (1:5000, 1:5000, 1:3000, 1:5000 and 1:5000, respectively, in 0.2% BSA-PBS) (Thermo Scientific, Roskilde, Denmark) at 100 μL/well for 20 min. The color was developed by adding the horseradish peroxidase substrate, 3,3′,5,5′-tetramethylbenzidine (TMB) (3.30, 5.50; Sigma Aldrich) at 100 μL/well and incubating the plates for 10 min, 15 min, 15 min, 15 min and 10 min, respectively, at room temperature in the dark. Further reaction was stopped with stop solution of 1 N H_2_SO_4_ at 50 μL/well. Finally, plates were read on an ELISA Multiscan Plus spectrophotometer (Spectra Max 250; Molecular Devices, San Jose, CA, USA) at 450 and 540 nm for IgM and IgG anti-PC as well as for IgM and IgG anti-MDA. For IgG1, IgG2, and IgA (anti-PC and anti-MDA with the Biotek 800 TS absorbance reader at 450 and 630 nm. All samples were measured in duplicate within a single assay and the coefficient of variation between the duplicates was < 15% for all the antibodies. Pooled serum from Sigma Aldrich (St Louis, MO, USA) was used as a standard control for each plate.

#### Statistics

Samples were tested using Student’s paired T test when normally distributed, as determined by Skewness and Kurtosis, if not normally distributed, values were compared using Wilcoxon signed rank test by using GraphPad Prism version 9.0.0 for Mac OS X, GraphPad Software, San Diego, California USA, www.graphpad.com.

## Results

Bears had expected metabolic changes, including increased cholesterol, triglyceride levels, glucose, insulin and cortisol levels, increased creatinine reflecting anuria, and decreased levels of uric acid, urea, ASAT and ALAT during hibernation. Laboratory results obtained in winter and summer are presented in Table 1.

**Table 1:** Baseline characteristics of bears in summer and winter.

Parameters	Summer	Winter	p value
Age (years)*	2–3	2–3	–
Weight (kg)**	40 (29–49)	33 (30–40)	0.07
Albumin (g/L)**	29.6 (24.4–31.0)	36.2 (35.4–37.3)	< 0.001
Triglycerides (mmol/L)**	1.9 (1.3–2.5)	3.7 (3.1–3.8)	< 0.001
Cholesterol (mmol/L)*****	5.9 (4.8–7.2)	11.3 (10.6–15.5)	< 0.01
Glucose (mmol/L)*	5.4 (4.1–6.3)	7.7 (5.6–8.1)	< 0.05
Iso-Insulin (mU/L)***	1.2 (0.7–1.8)	1.8 (1.3–2.2)	< 0.01
Cortisol (mmol/L)*****	88 (62–131)	348 (306–455)	< 0.01
Creatinine (µmol/L)*	74 (63–821)	203 (186–2310)	< 0.0001
Urea (mmol/L)*	12.1 (5.8–14.4)	2.9 (1.0–5.6)	< 0.002
Uric Acid (µmol/L)**	118 (100–133)	65 (59–76)	< 0.0001
ASAT (U/L)****	90 (73–137)	53 (41–56)	< 0.0055
ALAT (U/L)*****	44 (31–56)	12 (10–13)	< 0.0002
Osmolarity******	268 (266–276)	266 (264–274)	0.72

*12 pairs of bear sample taken for analysis.

**11 pairs of bear sample taken for analysis.

**9 pairs of bear sample taken for analysis.

****8 pairs of bear sample taken for analysis.

*****5 pairs of bear sample taken for analysis.

******4 pairs of bear sample taken for analysis.

**Figure 1:**
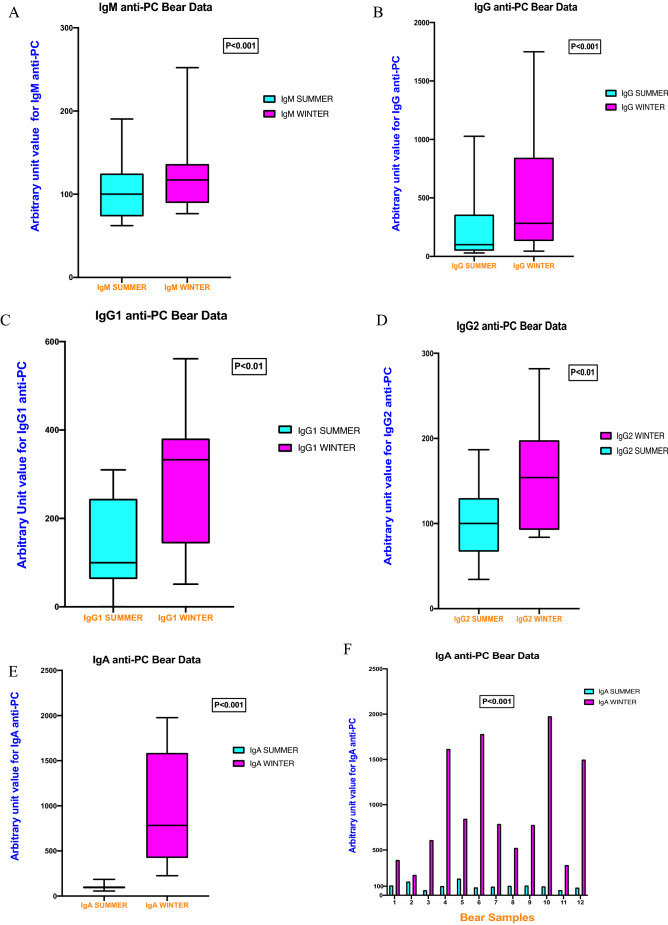
(**A**) IgM anti-PC levels during summer and winter (p < 0.001), (**B**) IgG anti-PC levels during summer and winter (p < 0.001), (**C**) IgG1 anti-PC levels during summer and winter (p < 0.01), (**D**) IgG2 anti-PC levels during summer and winter (p < 0.01), (**E**) IgA anti-PC during summer and winter (p < 0.001). (**F**) Individual values of IgA anti-PC for each bear during summer and winter (p < 0.001). Samples were tested using Student’s paired T test when normally distributed, as determined by Skewness and Kurtosis. if not normally distributed, values were compared using Wilcoxon signed rank test by using GraphPad Prism version 9.0.0 for Mac OS X, GraphPad Software, San Diego, California USA, www.graphpad.com.

Data are shown for IgM, IgG, IgG1, IgG2 and IgA anti-PC in Fig. 1. We have measured the unit values for each sample according to the equation:

$$Arbitrary \, unit \, value = \, \left( {\left( {Average \, optical \, density \, \left( {OD} \right) \, at \, 450\;{\text{nm }}{-} \, Average \, blank \, OD \, at \, 450\;{\text{nm}}} \right)/\left( {Median \, OD \, value \, of \, summer \, samples \, at \, 450\;{\text{nm}}} \right)} \right) \, \times \, 100.$$

When determined as arbitrary units (AU) with median set at 100 at summer marked and significant differences were observed between summer and winter for IgM anti-PC; 100 IQR (73.3–124.9) vs 117.2, IQR (89.4–136.4; p < 0.001), IgG anti-PC; 100 IQR (47.6–358.0) vs 282.6, IQR (131.7–844.2; p < 0.001), IgG1 anti-PC; 100 IQR (62.8–244.7) vs 333.0 IQR (143.4–380.8; p < 0.01), IgG2 anti-PC; 100 IQR (66.9–130.0) vs 153.94, IQR (92.4–198.0; p < 0.01) and IgA anti-PC; 100 IQR (85.9–107.9) vs 782.3, IQR (422.8–1586.0; p < 0.001).

**Figure 2:**
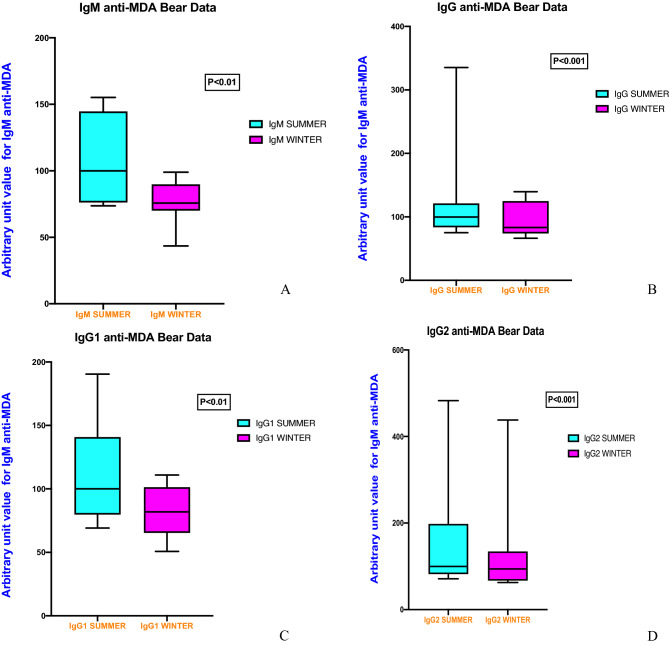
(**A**) IgM anti-MDA levels during summer and winter (p < 0.01), (**B**) IgG anti-MDA levels during summer and winter (p < 0.001), (**C**) IgG1 anti-MDA levels during summer and winter (p < 0.01), (**D**) IgG2 anti-MDA levels during summer and winter (p < 0.001). Samples were tested using Student’s paired T test when normally distributed, as determined by Skewness and Kurtosis. if not normally distributed, values were compared using Wilcoxon signed rank test by using GraphPad Prism version 9.0.0 for Mac OS X, GraphPad Software, San Diego, California USA, www.graphpad.com.

Data are shown for IgM, IgG, IgG1 and IgG2 anti-MDA in Fig. 2. When determined as arbitrary units (AU) with median set at 100 at summer marked and significant differences were observed between summer and winter for IgM anti-MDA; (100 IQR (76.1–144.6) vs 75.7, IQR (70.0–89.9; p < 0.01)), IgG anti-MDA; 100 IQR (83.6–121.3) vs 83.2, IQR (73.8–125,0; p < 0.001)) IgG1 anti-MDA; 100 IQR (79.7–140.9) vs 81.9, IQR (65.3–101.3; p < 0.01) IgG2 anti-MDA; 100 IQR (81.9–198.1) vs 94.0, IQR (67.0–134.5; p < 0.001).

## Discussion

We report that in hibernating brown bears anti-PC levels are significantly higher in the sedentary winter period compared to the active summer period. This was evident for all isotypes and subclasses of anti-PC studied, but more pronounced for IgA and IgG1. In contrast, both the innate and acquired cellular and humoral immune defences decrease during hibernation^5^ and anti-MDA showed a different pattern and IgA anti-MDA was not detectable. We confirm high cholesterol and triglyceride levels, increased glucose, insulin creatinine and cortisol levels and decreased levels of uric acid, urea, ASAT and ALAT during hibernation^1,3–5^.

Our observations may have several implications. At first, the high levels of anti-PC during the vulnerable hibernation period could contribute to protection against atherosclerosis and risk factors associated with this including dyslipidemia, insulin resistance and renal failure; three established human pro-atherogenic conditions^1,3,4,6^. In the human setting, anti-PC associate with protection against atherosclerosis, risk of CVD and mortality in CKD^6,13,15–25^ The associations may reflect underlying protective mechanisms, as indicated by several lines of evidence. Potential mechanisms include an anti-inflammatory effect by IgG anti-PC with inhibition of the effects of inflammatory phospholipids, with PC as the central agent^17^. Both IgM and IgG1 anti-PC increase the clearance of dead and dying cells that accumulate in atherosclerosis^26,31^. Anti-PC could also be atheroprotective by inhibition of uptake of OxLDL by macrophages, which then develop into atherogenic and inert foam cells^32^, and by inhibition of cell death caused by lysophosphatidylcholine; an important OxLDL component^29^. IgM anti-PC promote polarization of T regulatory cells, from atherosclerotic plaques, another immunomodulatory and anti-inflammatory property of anti-PC with direct relevance for the present study^33^. Moreover, IgG1, more than IgG2, anti-PC has protective properties associated with less atherosclerosis progress, less vulnerable plaques and mortality in CKD^29,30,34^ and IgA anti-PC is associated with favorable atherosclerosis progress^29^. It is likely that IgA anti-PC also shares at least some of these protective properties with other anti-PC subclasses and isotypes but more studies on IgA anti-PC are warranted. We also report that anti-MDA differs from anti-PC patterns among bears during summer and hibernation. The most striking difference is for IgA, which could not be detected for anti-MDA (while IgA anti-PC was much higher during hibernation). The other isotypes and subclasses tested were lower during hibernation. This unexpected finding indicates that changes in anti-PC is not a general reflection of increasing levels antibodies during hibernation. This observation strengthens the hypothesis that environmental factors related to infectious agents and microorganisms could play a role, since PC in contrast to MDA is a pathogen associated molecular pattern present on many pathogens^6,8,10,11^. In accordance, the rise in IgA anti-PC was the strongest, while IgA anti-MDA could not be detected. In a previous study, we determined that IgG2 anti-MDA is negatively associated with mortality in CKD, in contrast to all anti-PC isotypes and subclasses^28^.

Immune responses are usually characterized by an initial increase in IgM, followed by IgG increase after isotype switch and T-cell help, and then a decrease in levels of IgM. It is thus probable that IgM anti-PC is increased transiently in autumn and early in hibernation, and then may, followed by a prominent IgG response, with expansion and activation of memory B cells. If some of the protective properties are present only in IgM (not yet known), this could play a role in the earlier phases of hibernation. Regulation of circulating IgA probably follows a similar pattern as IgG^35^. It is likely that secretory, mucosal IgA anti-PC is produced also, to combat infectious agents, though this cannot easily be measured, at least not in this type of study on free-ranging animals. Microorganisms have developed mechanisms to counter IgA, including interference with its FC-receptor, which illustrates the importance of IgA in the defense against infections^35^. Thus, it could be speculated that IgA may contribute to the capacity of bears to heal infected wounds during hibernation^36^.

A number of other cardioprotective mechanisms may also be operative in hibernating bears. In American black bears a suppression of the intrinsic (but not extrinsic) pathway in the clotting cascade has been reported^37^. Although this mechanism could protect against blood clots it may not protect against lipid deposition in the arterial wall. Changes in serum proteins during hibernation may also be protective during the vulnerable hibernation period including increased capacity for bone maintenance and wound healing^38^. Other cardiac adaptions during hibernation, such as decreased functional measures of myocardial velocities^39^ may also be operative. Finally, a metabolic switch that shunts choline to generate betaine instead of the pro-atherogenic toxin Trimetylamine N-oxide (TMAO) during hibernation could hold clues for novel treatment options in burden of lifestyle diseases^40^.

The difference between antibody levels in summer and winter observed in this study provide clues of how these antibodies are induced and regulated. Anti-PCs were previously described as natural antibodies, being germ line encoded in laboratory mice, where one clone, T15, dominates. However, in humans this does not appear to be the case. We could not detect such a dominating clone, instead, human anti-PC showed signs of somatic mutation with Ig-switch and in addition, anti-PC are T cell dependent in humans^26,41^. In accordance, our recent finding that while humans are born with very low levels of anti-PC, during the first 2 years of life these are slowly rising, but still not at par with the mothers´ anti-PC levels. We interpreted this data as pointing to an important role played by environmental factors, especially the microbiome, but that genetic programs also may play a role. In contrast, anti-MDA was present at intermediate levels from birth and reached mothers’ levels after 2 years^42^ Of note, in this study, the bears were rather young (2–3 years) and the development of anti-PC during maturation in bears is not known. We cannot exclude that anti-PC is not fully developed at this age, and studies of anti-PC in older bears are warranted. However, there are advantages of studying younger bears, including reduced risk of potential confounders as pregnancy, sexual activity, and past diseases^5^. Other environmental factors, including diet cannot be excluded, but appear to be relatively weak in human^43^. However, for natural reasons, the enormous energy intake in bears with sometimes a doubling in weight with energy stored as fat, has no comparison to the human situation. It is not known if eating habits alone could influence antibody levels, even though our previous studies do not point to an important role played by diet in regulation. An interesting possibility with implications for human disease is the bears extreme consumption of berries in late summer and autumn in preparation for winter sleep^1^. Indeed, bilberries improve cardiometabolic function in a high risk population^44^, and consumption of anthocyanins associate with reduced risk of myocardial infarction in women^45^. Thus, the effect of bilberries on anti-PC levels in human risk populations need further studies.

Another possibility, the most likely in our opinion, is that infectious agents, i.e.; microorganisms of different kinds, play a role in stimulation of anti-PC in brown bears. Indeed, during hibernation, the profile of gut bacteria changes substantially, with reduced diversity and transplantation to germ free mice of microbiota from summer and winter indicate that the summer microbiota promote adiposity without impairing glucose tolerance^46^. PC is commonly exposed on bacteria as *S. pneumoniae* nematodes and parasites, where they bind to proteins or carbohydrates, and then to the immune system^11^. Even though PC when presented on oxidized lipids causes low grade inflammatory effects^12^, it may be anti-inflammatory and immune modulatory when presented on nematodes and parasites, where this may be a mechanism developed to evade the host´s immune response, in an arms race^11^. In brown bears, a high prevalence of zoonotic intestinal helminths and other parasites and nematodes has been reported^47–49^. Moreover, the prevalence of gastrointestinal parasites in grizzly bears were about double in the fall as compared to spring. Thus, bears are exposed to various infectious agents during the autumn, when energy consumption is extremely high. In previous studies, it has been proposed that brown bears void gastrointestinal parasites before hibernation, a notion supported by a previous study^50^. It is likely that the massive increase in anti-PC, not least IgA anti-PC, in the present study, contribute to this voiding^50^. The reasons for anti-MDA being lower in winter are not clear, neither the lack of IgA anti-MDA.

Individuals in Kitava, New Guinea, studied the 90’s, living as hunters, gatherers and horticulturalists did not have the burden of life-style diseases typical of the developed world. In Kitava, levels of anti-PC were much higher than among Swedish controls^51–53^. Our findings indicate that infections (not common in the Western world anymore) are a cause of their higher anti-PC levels. These observations led us to propose a development of the hygiene “old friends” hypothesis (where lack of exposure to infectious agents is believed to be of importance in diseases including asthma, typical of a modern lifestyle), where low levels of anti-PC is caused by lack of exposure to PC-bearing microorganisms including nematodes and parasites and bacteria as Treponema^51–53^. The present findings accord with this development of the hygiene/old friends hypothesis; i.e. low anti-PC could be described as an immune deficient state, predisposing to these types of conditions, where one common denominator is chronic inflammation.

The results of this short report should be considered with the following limitations. At first, we cannot exclude hemoconcentration contributed to the observed increase in anti-PC during hibernation. However, since plasma osmolarity did not differ between summer and winter, urea and uric acid levels decreased during hibernation and a different pattern for anti-PC and anti-MDA was observed this argues against that hemoconcentration had a major influence on our antibody analyses. Although we focused on anti-PC and anti-MDA, many other relevant antibodies including against other antigens on microorganisms could be of relevance. Still, PC as an antigen, and anti-PC as an immune-response is highly relevant, PC being both a DAMP and PAMP, and anti-PC representing a substantial portion of the circulating Ig. Also, anti-MDA is relevant since MDA is also a common antigen, recognized by the immune system and also interesting in comparison to PC, not being a pathogen associated molecular pattern.

We have not been able to determine the absolute values of circulating total IgG, IgG or IgA in bears which would have been interesting. However, in a previous study we determined different Ig via their constant regions. The levels of IGHM, IGHG1, IGLC1, IGKC, and IGJ were reduced in hibernation to about 90%. In contrast, the levels of IGHA1 which encodes a constant (C) segment of Immunoglobulin A heavy chain were doubled^5^. At least the increase during hibernation of IgG and IgM anti-PC is thus not present for these Ig:s in general. Still, it is possible that the increase in IgA anti-PC is paralleled also by general increases in other IgA specificities. Still, irrespective of cause of increase in anti-PC, this antibody has several protective properties, as discussed, and is also atheroprotective in animal models.

A larger study population would have been advantageous, and also blood sampling on more occasions, though this is not possible for ethical and logistic reasons. More functional studies on IgA in the circulation, including IgA anti-PC are needed. It would have been an advantage if second anti-bear antibodies would have been available, still, human and bear Ig are likely to show clear cross-reactivity, which is supported by previous studies of immunoglobulins in bears^5^.

Taken together, we report that anti-PC; especially IgG1 and IgA anti-PC, are strikingly high during hibernation in brown bears, while anti-MDA is low and IgA anti-MDA not detectable. We hypothesize that these changes contribute to the arterial protection of bears and their ability to withstand long and repetitive periods of dyslipidemia, kidney failure and insulin resistance during hibernation. Still, low anti-MDA could counteract such an effect, even though we suggest that the very high IgA anti-PC levels could outweigh this, one additional reason being that IgG2 anti-MDA may be detrimental^28^. Our observation could represent a natural immunization process preventing atherosclerosis and have therapeutic implications.
